# Phase I feasibility study of Olaparib in combination with loco-regional radiotherapy in head and neck squamous cell carcinoma

**DOI:** 10.1016/j.ctro.2023.100698

**Published:** 2023-11-04

**Authors:** Arash Navran, Abrahim Al-Mamgani, Hester Elzinga, Rob Kessels, Conchita Vens, Margot Tesselaar, Michiel van den Brekel, Rosemarie de Haan, Baukelien van Triest, Marcel Verheij

**Affiliations:** a Department of Radiation Oncology, Netherlands Cancer Institute, Amsterdam, The Netherlands; b Department of Head and Neck Surgery, Netherlands Cancer Institute, Amsterdam, The Netherlands; c Department of Biomerics, Netherlands Cancer Institute, Amsterdam, The Netherlands; d Department of Medical Oncology, Netherlands Cancer Institute, Amsterdam, The Netherlands; e Department of Head and Neck Surgery, Netherlands Cancer Institute and Department of Oral and Maxillo-Facial Surgery, Amsterdam University Medical Center, Amsterdam, The Netherlands; f Department of Radiation Oncology, Radboud University Medical Center, Nijmegen, The Netherlands

**Keywords:** PARP inhibitor, Olaparib, Radiosensitization, HNSCC, Radiotherapy, Feasibility, Maximum tolerated dose

## Abstract

**Purpose:**

PARP-inhibitors have potent radiosensitizing properties in pre-clinical models. To identify the maximum tolerated dose (MTD) of the PARP-inhibitor Olaparib in combination with radiotherapy in patients with head and neck cancer, a single institutional phase-I dose escalation trial was initiated.

**Patients and methods:**

The starting dose of Olaparib was 25 mg BID, combined with radiotherapy (70 Gy in 35 fractions). The MTD was defined as the highest dose-level at which not more than 20 % of patients experience dose-limiting toxicities (DLT) or as the highest reached dose in the absence of DLT’s.

**Results:**

One week Olaparib‐only treatment (25 mg QD) was administered to all patients prior to the start of radiotherapy. In dose-level I, Olaparib (25 mg BID) was combined with accelerated radiotherapy (70 Gy in 6 weeks). Because of DLT’s in 3 of the 4 treated patients (acute tracheotomy 5 and 7 months and osteoradionecrosis 7 months after treatment), the Olaparib dose was de-escalated to 25 mg QD, and combined with conventional radiotherapy (70 Gy in 7 weeks) (dose-level II). There were no DLT’s observed in 5 patients treated within dose-level II. After a median follow-up of 60 months, the 4-year LRC and OS rates were 77.8 % and 88.9 %, respectively.

**Conclusion:**

Olaparib 25 mg QD combined with conventionally fractionated radiotherapy was well tolerated and identified as the MTD while severe DLT’s were observed when Olaparib 25 mg BID was combined with accelerated radiation. This combination might be further explored in future Olaparib dose escalation studies in patients with locally-advanced HNSCC unfit for cisplatin-based chemoradiotherapy.

## Highlights

• We aim to identify the MTD of Olaparib in combination with RT in HNSCC.
• MTD of Olaparib is 25 mg QD, as no patient in this dose level developed DLT.
• Three of 4 the patients treated with Olaparib 25 mg BID developed DLT’s.
• Inclusion criteria for future Olaparib dose escalation trials to be refined.
• Patients with high risk profile & low risk of severe toxicity should be included.

## Introduction

The oncologic outcomes of patients with head and neck squamous cell carcinoma (HNSCC) has improved since the addition of cisplatin or cetuximab to the radiation treatment and/or the use of altered radiotherapy fractionation schedules[1], [2], [3], [4]. Despite these improvements, the loco-regional control (LRC) and overall survival (OS) in certain subgroups of HNSCC are still disappointing such as patients with high-risk profiles (locally-advanced disease, heavy smokers and/or HPV-negative tumor), patients who are unfit for cisplatin or cetuximab and those who are not able to receive a total dose of cisplatin ≥200 mg/m^2^ [5], [6]. In those patients, alternative treatment intensification is needed. Different treatment intensification strategies have been investigated to improve LRC in these patients, including the use of hypoxic modification, radiation dose escalation or the addition of neo-adjuvant immune checkpoint blockade [7], [8], [9], [10], [11], [12], [13], [14]. Another potential way to improve LRC in patients with HNSCC is by targeted radiosensitization, focusing on interference with DNA damage repair such as the addition of a PARP-inhibitor (PARPi). PARPi as single agent have shown potent anti-cancer activity in tumors with deficiencies in DNA repair mechanisms such as homologous recombination in different pre-clinical and clinical studies [15], [16], [17], [18], [19]. It has been hypothesized that, in the context of homologous recombination defect (HRD), PARPi in combination with radiotherapy might be effective in improving disease control in different tumor sites, including HNSCC [20], [21], [22], [23], [24]. Because of the limited data on the safety, tolerability and anti-tumor efficacy of the oral PARPi Olaparib as radiosensitizer in cancer patients, three phase I studies were designed and conducted in our institution to identify the maximum tolerated dose (MTD) of Olaparib in combination with radiotherapy in non-small cell lung cancer (NSCLC), breast cancer and HNSCC [25]. Based on our preclinical studies and clinical phase-I trial in NSCLC, it was concluded that the dose of Olaparib as radiosensitizer is a factor of 10 lower than the dose effective as single agent [21]. The aim of the current study is to report on dose-limiting toxicity (DLT) and to identify the MTD of Olaparib in combination with the standard of care 70 Gy of radiotherapy in patients with HNSCC.

## Materials and methods

### Study population and treatment

The current study was approved by our institutional medical ethics committee (ClinicalTrials.gov identifier: NCT02229656). For a detailed description of the study protocol we refer to the publication by de Haan et al. [25]. Briefly, patients with T1-2N0-2bM0 laryngeal cancer, HPV-negative oropharyngeal cancer or HPV-positive patients with a history of smoking ≥10 pack-years could be included (n = 5). Also patients with locally-advanced disease (T3-4, N2c-3) from both tumor sites unfit for concurrent cisplatin or cetuximab were eligible for inclusion. Four of the study patients were unfit for radiotherapy in combination with cisplatin or cetuximab because of age above 70 years (n = 1), poor renal function (n = 2) or peripheral vascular disease (n = 1). Pre-treatment evaluations consisted of chest X-ray, ultrasound with fine needle aspiration cytology (FNAC), and head and neck MRI or CT scan. In patients with locally-advanced disease (cT3-4,N2b-N3), 18-FDG-PET/CT was also performed. All patients were discussed at our weekly multidisciplinary head and neck tumor board. Based on the joint recommendations of the multidisciplinary board, eligible patients for the study were identified and asked for trial participation. All patients entered in the study (n = 12) signed the informed consent. However, three patients were not treated with Olaparib-radiotherapy but with radiotherapy alone or in combination with cisplatin or cetuximab, leaving 9 patients evaluable for the primary objective of the study.

All patients were treated with volumetric-modulated arc therapy (VMAT). The high-risk CTV was generated by adding 6 mm isotropic margin to the delineated GTV-P (primary tumor) and GTV-N (involved nodes) and subsequently edited to the adjacent non-involved bone and/or air. The elective low-risk CTV-P and CTV-N was generated by adding 6 mm margin to high-risk GTV. The elective low-risk CTV of the neck was defined as level I-V in case of node-positive and level II-IV in case of node-negative disease. The PTV included a margin of 3 mm around the CTV. The radiation dose to the high-risk PTV consists of 70 Gy and to the elective low-risk PTV 54.25 Gy in 35 fractions using simultaneous integrated boost. The radiation was given in 2 Gy per fraction, 6 fractions a week (accelerated schedule) in patients treated within dose-level I of the Olaparib (n = 4) and 5 fractions a week (conventional schedule) in patients treated in dose-level II of the Olaparib (n = 5).

### Olaparib

Treatment with Olaparib was started one week before start of radiotherapy (25 mg QD) and continued until 2 days after end of radiotherapy. Olaparib was administered orally. In dose-level I, 25 mg of Olaparib was given twice a day (BID) with 12-h interval and combined with accelerated radiation. In dose-level II, 25 mg of Olaparib was given once a day (QD) combined with conventional radiation.

### Objectives and endpoints of the study

Primary objective was to identify the MTD of Olaparib in combination with radiotherapy. The MTD of Olaparib was defined as the highest dose-level at which not more than 20 % of patients experience DLT or as the highest reached dose in the absence of any DLT.

The primary endpoint was the incidence of acute (i.e. within 90 days after treatment) and late (i.e. beyond 90 days until one year after treatment) DLT. These toxicities were reported using Common Terminology Criteria for Adverse Events, version 4.0. Secondary endpoints (feeding tube dependency at 1 year, LRC, and OS were also reported). The toxicity type and grade (grade 1–5) were collected by the trial personals from the case report form (CRF) of all participating patients.

According to the study protocol, non-hematological toxicities regarded as DLT in the acute phase were grade ≥4 mucositis, dysphagia, dermatitis, grade ≥3 hemorrhage, aspiration, trismus, grade ≥3 laryngeal edema (only in oropharyngeal cancer), and discontinuation of radiotherapy >3 fractions and cumulative discontinuation of Olaparib for >20 % of the total prescribed dose due to toxicity. DLT’s in the late phase were recorded if any one of the following toxicities were observed: grade ≥4 dysphagia or aspiration, tracheotomy in the absence of any evidence of tumor recurrence, grade ≥3 hemorrhage, skin atrophy, trismus, osteoradionecrosis, radiation dermatitis, pneumonitis, grade ≥3 laryngeal stenosis (only in oropharyngeal cancer), grade ≥2 fistula or mucosal ulcer persists ≥6 months after treatment, fibrosis limiting joint or orifice movement (e.g. mouth) and/or limiting self-care ADL.

### Follow-up

During treatment patients were seen twice weekly by the radiation oncologist and medical oncologist in order to monitor and register acute toxicities. After completion of treatment, patients were seen every week until the acute toxicity had subsided. Three months after treatment, the response evaluation was done by clinical examination, including flexible naso-endoscopy, CT scan or MRI, ultrasonography and FNAC, if indicated. Thereafter, patients were followed up 2-monthly in the first year, 3-monthly in the second year and 6-monthly thereafter until 5 years.

## Results

Table 1 shows the baseline patients characteristics. All patients were male and the median age was 66 years (range; 48–74). All patients, except one (89 %) had a laryngeal cancer. Four patients were treated in dose-level I where Olaparib 25 mg BID was combined with accelerated radiotherapy in 6 weeks. Because of the occurrence of DLT’s in 3 of these patients, the study team decided to de-escalate the treatment to conventional radiotherapy in 7 weeks, combined with 25 mg of Olaparib QD.

**Table 1:** Baseline patients characteristics.

Follow-up time, in months	Numbers (%)
Median	60.06
Range	31.8–79.6
Gender	
Male	9 (100 %)
Age, in years	
Median	66
Range	48–74
Tumor site	
Larynx	8 (89 %)
Oropharynx	1 (11 %)
T-stage	
T2	5 (56 %)
T3	4 (44 %)
N-stage	
N0	5 (56 %)
N1	1 (11 %)
N2b	2 (22 %)
N2c	1 (11 %)
AJCC stage	
II	3 (33 %)
III	3 (33 %)
IV	3 (33 %)
Smoking history	
Never	1 (11 %)
>10 pack years	8 (89 %)
Smoking at diagnosis	
Yes	6 (66 %)
No	3 (33 %)
Resume smoking after RT	
Yes	2 (22 %)
No	7 (78 %)
RT scheme	
Accelerated	4 (44 %)
Conventional	5 (56 %)
Dose Olaparib	
25 mg twice a day	4 (44 %)
25 mg once a day	5 (56 %)

Abbreviations: AJCC: American Joint Committee on Cancer; RT: radiotherapy.

Table 2 shows the acute and late treatment-related toxicities, including the DLT’s. No grade ≥4 acute toxicity was observed. All patients experienced one or more grade 1–3 acute and/or late toxicities.

**Table 2:** Acute and late radiation-related toxicity including the dose-limiting toxicity reported (marked with *).

	Number (%) of events
Acute toxicity	
Grade 2 dermatitis	5 (56 %)
Grade 3 dermatitis	4 (44 %)
Grade 2 mucositis; all types	1 (11 %)
Grade 3 mucositis; all types	8 (89 %)
Grade 2 dysphagia	5 (56 %)
Grade 3 dysphagia	4 (44 %)
Grade 2 oral and/or pharyngeal pain	6 (66 %)
Grade 3 oral and/or pharyngeal pain	3 (33 %)
Grade 2 xerostomia	9 (100 %)
Grade 1 dysguesia	7 (78 %)
Grade 2 dysguesia	2 (22 %)
Grade 2 fatigue	4 (44 %)
Grade 2 dyspnea	2 (22 %)
Grade 1 voice alteration	2 (22 %)
Grade 2 voice alteration	7 (78 %)
Grade 1 laryngeal edema	8 (89 %)
Grade 2 aspiration	5 (56 %)

Late toxicity	
Grade 1 dysphagia	2 (22 %)
Grade 2 dysphagia	4 (44 %)
Grade 3 dysphagia	2 (22 %)
Grade 1 xerostomia	2 (22 %)
Grade 2 xerostomia	7 (78 %)
Grade 1 laryngeal edema	1 (11 %)
Grade 2 laryngeal edema	1 (11 %)
Grade 3 laryngeal edema	2 (22 %)
Grade 1 aspiration	2 (22 %)
Grade 3 aspiration	2 (22 %)
Grade 4 dyspnea/tracheotomy*	2 (22 %)
Grade 2 penumonitis	2 (22 %)
Grade 2 fatigue	1 (11 %)
Grade 3 mandibular osteoradionecrosis*	1 (11 %)
Garde 2 trismus	1 (11 %)
Grade 2 skin atrophy	1 (11 %)

Three of the four patients treated in dose-level I developed DLT’s. Characteristics of those patients with any event (DLT or tumor recurrence) are shown in Table 3.

**Table 3:** Characteristics of patients with any events (dose-limiting toxicity or oncologic event).

	#P1	#P2	#P3	#P8	#P9
Age	54	68	51	66	73
Gender	M	M	M	M	M
Tumor site	Larynx	Larynx	Oropharynx	Larynx	Larynx
TNM classification	T3N1	T3N0	T2N2b	T2N2c	T3N2b
Dose olaparib	25 mg BID	25 mg BID	25 mg BDI	25 mg QD	25 mg QD
Radiation scheme	Accelerated	Accelerated	Accelerated	Conventional	Conventional
WHO status before treatment	0	0	0	0	0
ACE-27 score	2	1	1	2	1
Smoking history	40 pack years	15 pack years	30 pack years	20 pack years	30 pack years
Active smoking at disgnosis	Yes	Yes	Yes	Yes	No
Active smoking during treatment	No	No	No	No	No
Smoking status after treatment	Resume smoking	Stop	Resume smoking	Stop	Stop
DLT	Tracheotomy	Tracheotomy	ORN mandible	No	No
Time from end RT to DLT (months)	5	7	7	NA	NA
Local and/or regional failure	NED	NED	NED	LRF	RF
Time from end RT to failure (months)	NA	NA	NA	7	20
Alive at last follow-up	No	No	No	Yes	Yes
Time end RT to death (months)	52	31	79	NA	NA

Abbreviations: #P1: refer to patient study number; M: male; TNM tumor node metastasis; BID: twice a day; QD: once a day; ACE-27: adult comorbidity evaluation; DLT: dose-limiting toxicity; ORN: osteoradionecrosis; RT: radiotherapy; NA: not applicable; NED: no evidence of disease; LRF: loco-regional failure; RF: regional failure.

**Fig. 1:**
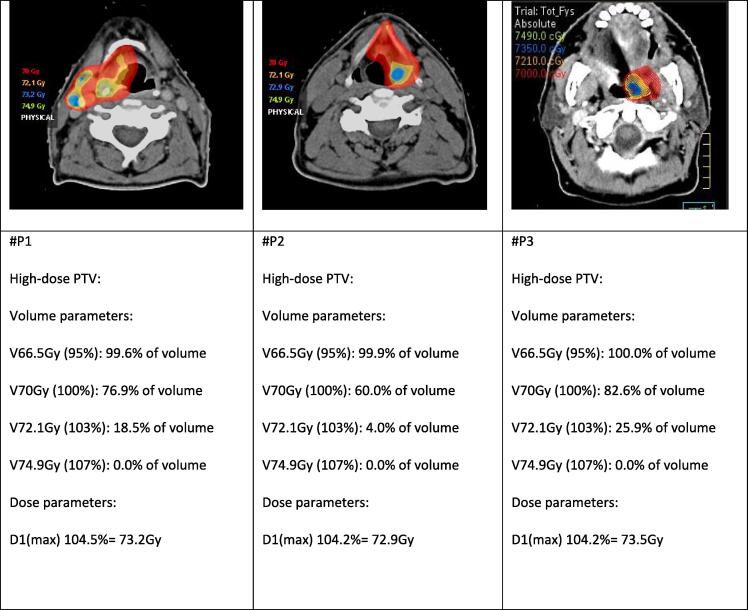
The dose distribution of the 3 patients with dose-limiting toxicity. The first and second study patient (#P1 and #P2) who developed grade 4 dyspnea as DLT because of laryngeal stenosis requiring acute tracheotomy and the third study patient (#P3) who developed mandibular osteoradionecrosis as DLT show no overdosage or hotspots at the adjacent laryngeal cartilage or the mandible. Only high-dose areas were illustrated: red = 70 Gy; orange = 72.1 Gy (103 % of the prescribed dose), no any voxel received ≥ 107 % of the prescribed dose, and blue = D_max_. (For interpretation of the references to colour in this figure legend, the reader is referred to the web version of this article.)

With regard to the DLT’s, the first and second study patient developed grade 4 dyspnea due to laryngeal stenosis requiring acute tracheotomy at 5 and 7 months after treatment, respectively. In the first patient cordectomy was done 3 years after treatment because of repeated aspiration and persistent swallowing problems. The third study patient with oropharyngeal cancer developed osteoradionecrosis of the mandible 7 months after treatment, treated conservatively with repeated sequestrectomy and hyperbaric oxygen without satisfactory results and ended up with a segmental mandibulectomy and fibula reconstruction. Table 4 and Fig. 1 show different dose and volume histogram parameters of the three patients with DLT’s. There were no hotspots or overdosages seen on the radiation plan in the neighborhood of the adjacent laryngeal cartilage or the mandible of the three patients with DLT’s. Two of the three patients who developed DLT resumed smoking after finishing radiotherapy. Only one patient was feeding tube dependent at 1 year after treatment. No patient treated in dose-level II developed a DLT.

**Table 4:** Dose and volume historgram parameters of the three patients with dose-limiting toxicity.

	#P1	#P2	#P3
Tumor site	Larynx	Larynx	Oropharynx
Type of DLT	Tracheotomy	Tracheotomy	ORN mandible
Dmean larynx	64.1 Gy (91.6 % of PD)	62.6 Gy (89.4 % of PD)	not relevant
Dmax larynx	73.5 Gy (104.9 % of PD)	73.1 Gy (104.4 % of PD)	not relevant
D1 (larynx) (1 % volume)	72.7 Gy (103.9 % of PD)	71.9 Gy (102.8 % of PD)	not relevant
Dmean mandible	not relevant	not relevant	38.8 Gy (55.4 % of PD)
Dmax mandible	not relevant	not relevant	72.7 Gy (103.8 % of PD)
D1 (mandible) (1 % volume)	not relevant	not relevant	71.2 Gy (101.6 % of PD)
Dmean oral cavity	not relevant	not relevant	43.1 Gy (61.5 % of PD)
Dmax oral cavity	not relevant	not relevant	74.8 Gy (106.8 % of PD)
D1 (oral cavity) (1 % volume)	not relevant	not relevant	72.5 Gy (103.6 % of PD)

Abbreviations: #P1: refer to patient study number; DLT: dose-limiting toxicity; ORN: osteoradionecrosis; PD: prescribed dose; D1 means the dose reported in 1 % of the volume of that specific organ at risk. None of the relevant organs at risk received ≥107 % of the prescribed dose. Not relevant means that these dose parameters are not relevant for that specific DLT. In P#1 and #P2 the dose parameters of the mandible and oral cavity are not relevant for the development of dyspnea and need for tracheotomy as DLT while in #P3 the dose parameters of the larynx are not relevant for the development of ORN of the mandible.

**Fig. 2:**
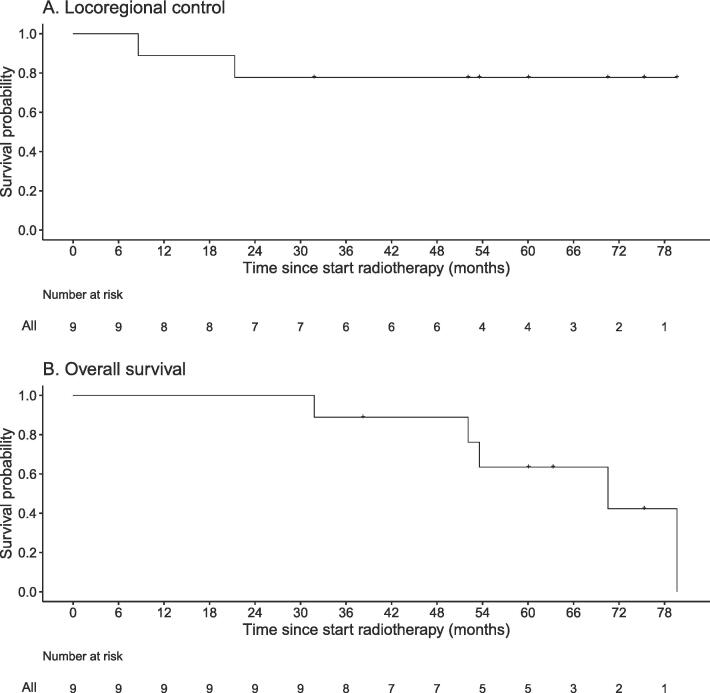
Kaplan-Meier curves of loco-regional control and overall survival.

With regard to the anti-tumor efficacy of the combined treatment, no patient treated in dose-level I developed any type of failure, while two patients treated in dose-level II developed loco-regional failure and regional failure 7 and 20 months after treatment, respectively. The patient with loco-regional failure was treated with total laryngectomy and neck dissection and is still alive at the time of last follow-up with no evidence of disease, 4.5 year after the salvage surgery. The patient with regional failure presented with an irresectable neck recurrence and treated with 3-weekly permbrolizumab with a very good response (nearCR) and is still alive without progression, 17 months after the recurrence. None of the patients developed distant metastasis. After a median follow-up of 60 months (range; 31.8–79.6), the 4-year LRC and OS rates were 77.8 % (95 %CI 54.9 % – 100 %) and 88.9 % (95 %CI 70.6 % – 100 %), respectively (Fig. 2). The median survival of the whole group is 70.5 months. No patient died because of the index HNSCC; two patients died because of second primary lung cancer, one because of second primary tongue cancer, and two because of comorbidity 69, 79, 45, 52, and 31 months after treatment of the index HNSCC, respectively.

## Discussion

This phase-I study aims to identify the MTD and to investigate the feasibility and safety of Olaparib in combination with radiotherapy in patients with HNSCC. We showed that Olaparib 25 mg QD combined with a conventional scheme of radiotherapy was well tolerated and safe as there were no grade ≥ 4 acute toxicity and no DLT reported while severe DLT’s were observed when Olaparib 25 mg BID was combined with accelerated radiation. Hence, Olaparib 25 mg QD was regarded as the MTD. Two of three patients who developed DLT have resumed smoking after treatment. Although in none of these patients overdosage was seen on the radiation plan, attention should be paid during the treatment planning to avoid hotspots at adjacent cartilages and/or bones in order to minimize the risk of the development of chondro- or osteoradionecrosis.

The efficacy of Olaparib monotherapy has been demonstrated in different tumor sites such as metastatic breast and ovarian cancer [15], [16], [17], [18]. Recently, PARPi have shown potent anti-tumor activity not only in those settings attributed to BRCA mutations but also in tumors with HRD [26], [27], [28]. Heitmann et al. [28] demonstrated that PARPi have a potent anti-tumor effect in a subset of HNSCC cell lines where HRD was present. Different prospective clinical studies have been initiated to investigate the tolerability and/or efficacy of PARPi in HNSCC either alone or in any combination with radiotherapy, cetuximab (NCT01758731), cisplatin (NCT02308072), and/or anti-PD1 durvalumab (NCT02882308). With regard to the combination of Olaparib and radiotherapy the data is scarce. In preclinical studies, synergistic activity between cetuximab and PARPi has been demonstrated in several HNSCC cell lines [29]. Different pre-clinical studies have shown that Olaparib is a potent radiosensitizer as well [30], [31]. Therefore, the group of Raben from the University of Colorado has investigated the safety and toxicity of combining Olaparib with cetuximab and a conventional scheme of radiotherapy for patients with locally-advanced HNSCC and heavy smoking history. Three out 16 patients developed DLT; two patients with grade 4 dermatitis (both received Olaparib 100 mg BID) and one with grade 3 nauseas and vomiting at Olaparib 200 mg BID. All these patients were treated conservatively with satisfactory results. The MTD in that study was determined to be 50 mg BID. However, the recommended phase II dose was 25 mg BID. After a median follow-up of 26 months the median survival was 37 months [32]. This dose level of 25 mg BID in combination with a conventionally fractionated scheme of radiation is worthy to be tested in future studies in patients who are at high risk of disease recurrence but are not suitable for cisplatin-based chemoradiation.

Also in other tumor sites, different studies aimed to identify the MDT of Olaparib in combination with radiotherapy. In patients with NSCLC this combination was also investigated in our institution. The MTD in those patient was 25 mg QD, as 2 of 7 patients treated with 25 mg BID experienced 3 late-onset DLT’s (esophageal and pulmonary) while only one of the 11 patients treated with 2 5 mg QD developed grade 3 pneumonitis as DLT. The 2-year LRC-rates was 84 % in patients where the Olaparib -radiotherapy schedule was combined with cisplatin and 83 % in those treated without cisplatin. The median survival was 28 months at median follow-up time of 4.1 years [21]. The combination of Olaparib and radiotherapy in patients with inoperable and/or metastatic breast cancer with indication for breast irradiation was also investigated in our institution (NCT02227082) as part of three parallel phase I-trials [25] aiming to identify the MTD in NSCLC, HNSCC (The current study) and breast cancer. The results of our breast cancer trial will be published soon. The RADIOPARP phase-I trial investigated the DLT and MTD in 24 patients with triple-negative breast cancer treated with Olaparib and postoperative loco-regional radiotherapy. The MTD in this study was 200 mg of Olaparib BID, without any DLT reported [20].

The MTD in HNSCC and NSCLC studies in our institution was 25 mg of Olaparib QD. Even with such low dose of Olaparib as compared to its use as single agent, effective radiosenitization was achieved as, at the MTD, Olaparib reduced PAR levels by more than 95 % and abolished radiation-induced PARylation ex vivo [33]. The LRC and OS rates of both studies were promising. However, no solid conclusions about the oncologic efficacy could be drawn because of the small sample size in both studies. Although higher doses of Olaparib might further improve the oncologic outcomes, the most important limiting factor in escalating the dose of Olaparib in the current study is the development of severe DLT’s in patients treated in dose-level I (25 mg BID). This means that the selection criteria for future Olaparib dose escalation studies in HNSCC with dose >25 mg QD should be refined to include patients at high risk of disease recurrence and at lower risk of radiation-related toxicity. Patients with high-risk profiles (locally-advanced disease, heavy smokers and/or HPV-negative tumor) unfit for cisplatin-based chemoradiation and who are willing to stop smoking before treatment might be appropriate candidates for such studies, especially in tumors not too close to bony structures, in order to minimize the risk of chondro- or osteoradionecrosis especially when accelerated radiotherapy schemes are applied. Different studies have shown that cumulative dose of cisplatin ≥200 mg/m^2^ is an important cut-off dose level below which the oncologic outcomes of patients deteriorate, compared to these who received at least 200 mg/m^2^ (3-year OS-rates were 74 % and 51 %, respectively; p < 0.0001) [6]. In that study, 25 % of all patients were not able to receive more than one course of cisplatin because of toxicity [6], [34]. These patients might also be candidates for concomitant Olaparib -radiotherapy when continuation of cisplatin is not possible because of toxicity.

Another potential option to fine-tune the dose of Olaparib in combination with radiotherapy is to add anti-PD1 immune therapy to the Olaparib-radiotherapy schemes, as anti-PD1 combined with radiotherapy was feasible and showed efficacy in HNSCC [14], [35]. The ongoing OPHELIA study (NCT02882308) where HNSCC patients are pre-operatively treated with PARPi and durvalumab shows promising preliminary results with 2 of 9 patients achieving pCR after neo-adjuvant treatment.

The authors are aware of the limitations of the current study. Evaluating the anti-tumor efficacy of Olaparib in combination with radiotherapy is limited by the small sample size. The radiotherapy scheme in patients treated in the Olaparib dose-level I (25 mg BID) was an accelerated scheme. Three of the 4 patients treated at that dose-level developed DLT. Accelerated radiation schemes are well known to improve oncologic outcomes but at the cost of increasing acute toxicity [36]. The accelerated scheme used in dose-level I might be the confounding factor because it is quite difficult to indicate whether Olaparib 25 mg BID was responsible for the increased toxicity in those patients or the accelerated radiation. This might be the reason why patients treated in the study of Olaparib combined with conventional scheme of radiotherapy and cetuximab [32] have reached the MTD of 50 mg BID.

In conclusion, Olaparib at 25 mg QD combined with a conventional scheme of radiotherapy was well tolerated and thus regarded as the MTD. The selection criteria for future Olaparib dose escalation trials need to be refined to include patients at high-risk of disease recurrence and lower risk of toxicity such as patients with locally-advanced HNSCC unfit for cisplatin and in those who are willing to stop smoking before treatment and preferably in tumors not too close to the mandible or laryngeal cartilage to avoid any hotspots or overdosage at these structure, as this might increase the risk of severe radionecrosis of these structures.

## CRediT authorship contribution statement

**Arash Navran:** . **Abrahim Al-Mamgani:** Writing – review & editing. **Hester Elzinga:** Writing – review & editing. **Rob Kessels:** Writing – review & editing. **Conchita Vens:** Methodology, Writing – review & editing. **Margot Tesselaar:** Methodology, Writing – review & editing. **Michiel van den Brekel:** Methodology, Writing – review & editing. **Rosemarie de Haan:** Writing – review & editing. **Baukelien van Triest:** Methodology, Writing – review & editing. **Marcel Verheij:** Methodology, Writing – review & editing.

## Declaration of Competing Interest

The authors declare that they have no known competing financial interests or personal relationships that could have appeared to influence the work reported in this paper.
